# Repetitive Microtrauma and Negative Ulnar Variance as Possible Culprits of Avascular Necrosis of the Lunate

**DOI:** 10.7759/cureus.5943

**Published:** 2019-10-19

**Authors:** Milad Heydari-Kamjani, Sadaf Afraz, Danay Herrera, Michelle Demory Beckler, Marc M Kesselman

**Affiliations:** 1 Osteopathic Medicine, Nova Southeastern University Dr. Kiran C. Patel College of Osteopathic Medicine, Fort Lauderdale, USA; 2 Immunology, Nova Southeastern University Dr. Kiran C. Patel College of Osteopathic Medicine, Fort Lauderdale, USA; 3 Rheumatology, Nova Southeastern University Dr. Kiran C. Patel College of Osteopathic Medicine, Fort Lauderdale, USA

**Keywords:** avascular necrosis, lunate, trauma, negative ulnar variance

## Abstract

Kienböck’s disease is characterized by avascular necrosis of the lunate. Its pathophysiology involves a complex interplay of repetitive microtrauma, anatomical and vascular variances. Early diagnosis of this entity is challenging as disease presentation is nonspecific and can mimic common wrist pathologies such as fractured or sprained wrist. Here we report a case presentation of Kienböck’s disease Stage IIIA in a 28-year-old healthy male. Initial radiographs of the left wrist were inconclusive and two weeks later the diagnosis was confirmed with a magnetic resonance imaging. The patient was initially treated with cast immobilization for four months but remained symptomatic with no improvements in pain or function. He then elected to participate in left radial shortening osteotomy with a vascularized bone graft from the distal radius. Unfortunately, both conservative and invasive procedures did not prevent end-stage disease characterized by the complete collapse of the lunate. However, 18 months post-surgical follow-up, the patient continues to remain pain-free with no limitations to his daily living activities.

## Introduction

Kienböck’s disease is a rare condition characterized by avascular necrosis of the lunate bone in the wrist, which results in the death of the bone. The condition commonly presents among individuals between the ages of 20 and 40 years [1]. While limited knowledge into the pathogenesis of Kienböck’s disease exists, multiple factors including repetitive microtrauma, anatomical variances, and/or vascular abnormalities have been suspected to contribute to the disease onset and progression. During trauma, ruptured blood vessels may decrease the delivery of nutrients to the lunate [2]. Anatomically, negative ulnar variance (UV), may increase the mechanical stress on the lunate exerted by a relatively long radius [3,4]. In addition, individuals with vascular abnormalities including a single blood vessel supply to the lunate are thought to be at a higher risk of developing avascular necrosis [5].

Diagnosis of Kienböck’s disease is commonly made based on physical examination and imaging. Disease presentation can be non-specific and mimic common wrist pathologies such as sprained or fractured wrists, which typically present with persistent dorsal wrist pain with reduced grip strength and range of motion [2]. Imaging modalities used for diagnosis include radiographs and magnetic resonance imaging (MRI). Plain film radiographs are most commonly used for the initial diagnosis but can often be inconclusive during earlier stages of the disease [6]. MRI is another option and commonly used when radiography is unclear. MRI tends to be more sensitive and can evaluate subtle initial changes such as inflammation, edema, and ischemia. Treatment of the condition is commonly directed by Lichtman’s classification system, which first developed by David Lichtman in 1977, and that takes into account radiographic appearances to guide treatment [7]. The goal of treatment is to prevent further collapse of the lunate, restore blood flow, reduce pain and delay osteoarthritis. There are various therapeutic approaches available, and selection depends on the stage of the disease directed by Lichtman’s classification system. Cast immobilization is the first-line treatment for earlier stages of the disease, despite its lack of efficacy in reversing disease progression [8]. More invasive approaches include lunate revascularization, lunate replacement with silicone prosthetic, and radial shortening or ulnar lengthening in the presence of negative UV [7,9,10]. Salvage procedures including proximal row corpectomy and wrist arthrodesis are preserved for end-stage Kienbock’s disease and are mostly aimed to reduce pain [11,12].

## Case presentation

**Figure 1:**
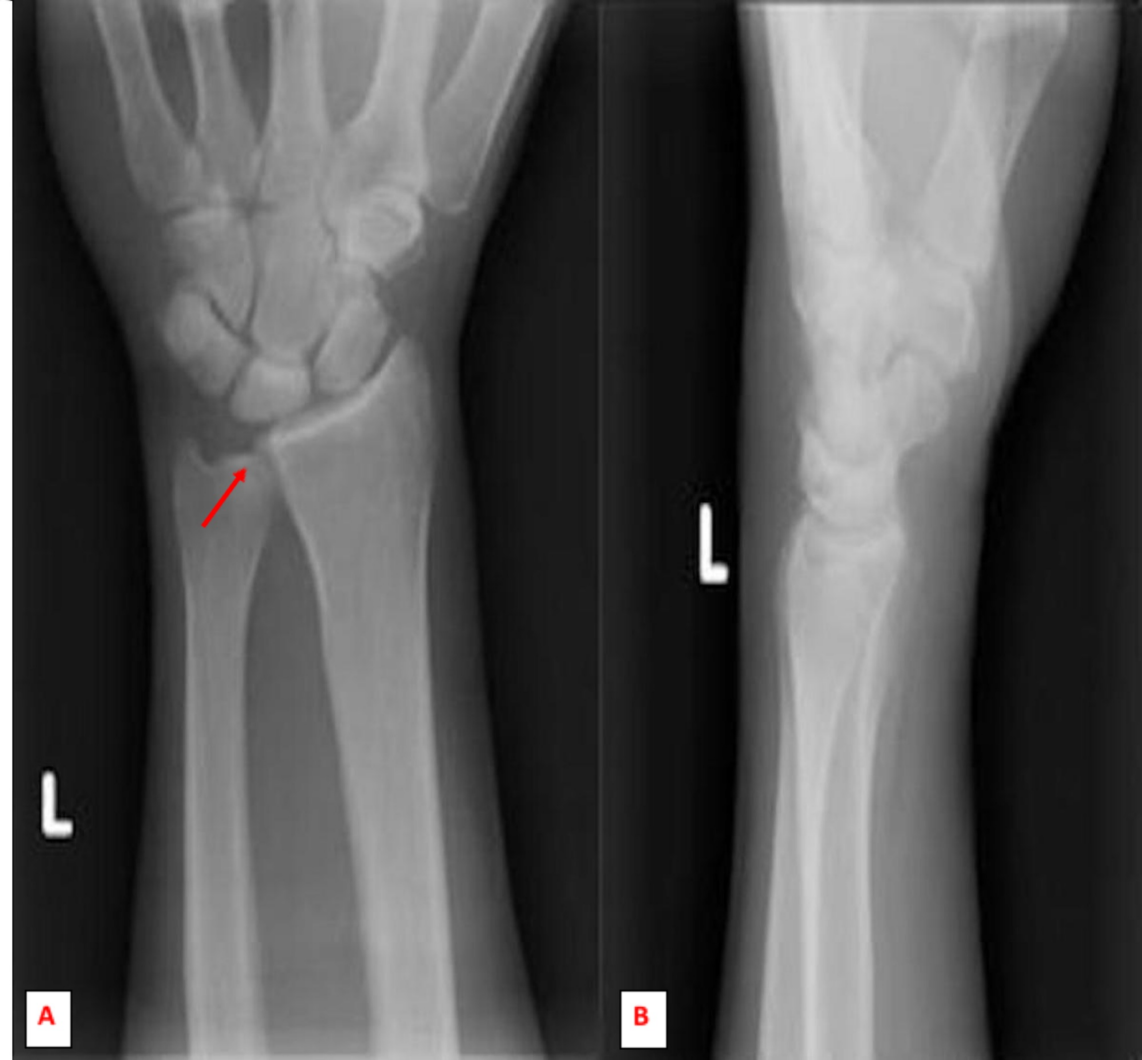
Initial anterior-posterior (A) and lateral (B) radiographic imaging of the left wrist showed normal alignment, articular surfaces and preserved spaces. No obvious lytic or blastic lesions. A negative ulnar variance noted.

**Figure 2:**
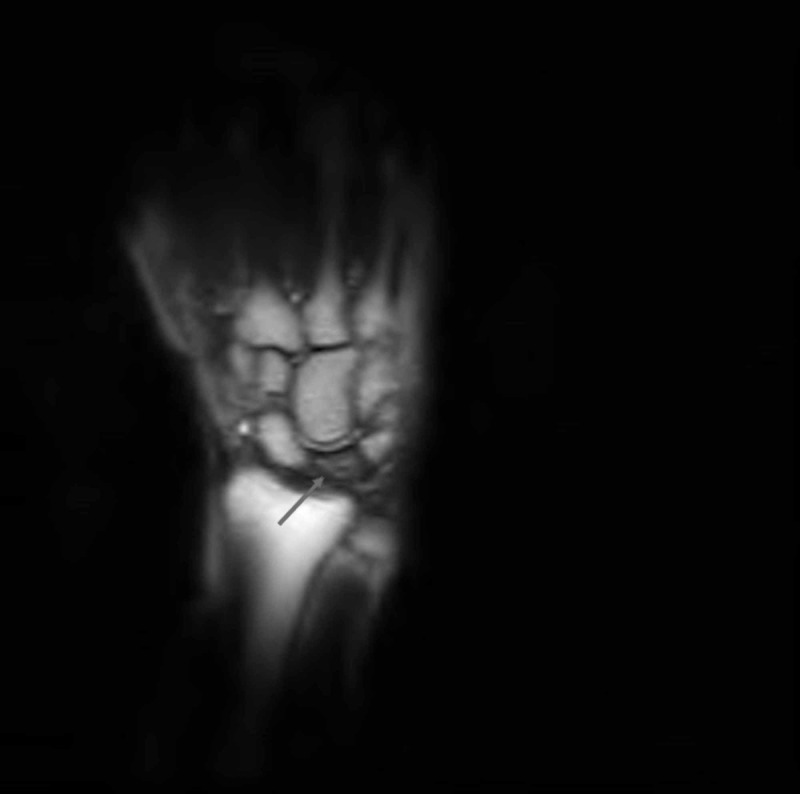
Coronal T1-weighted MR image of the left wrist revealed mild flattening of the lunate, a linear fissure fracture superiorly, and a decreased signal intensity of the lunate suggestive of Kienböck’s disease, stage IIIA.

**Figure 3:**
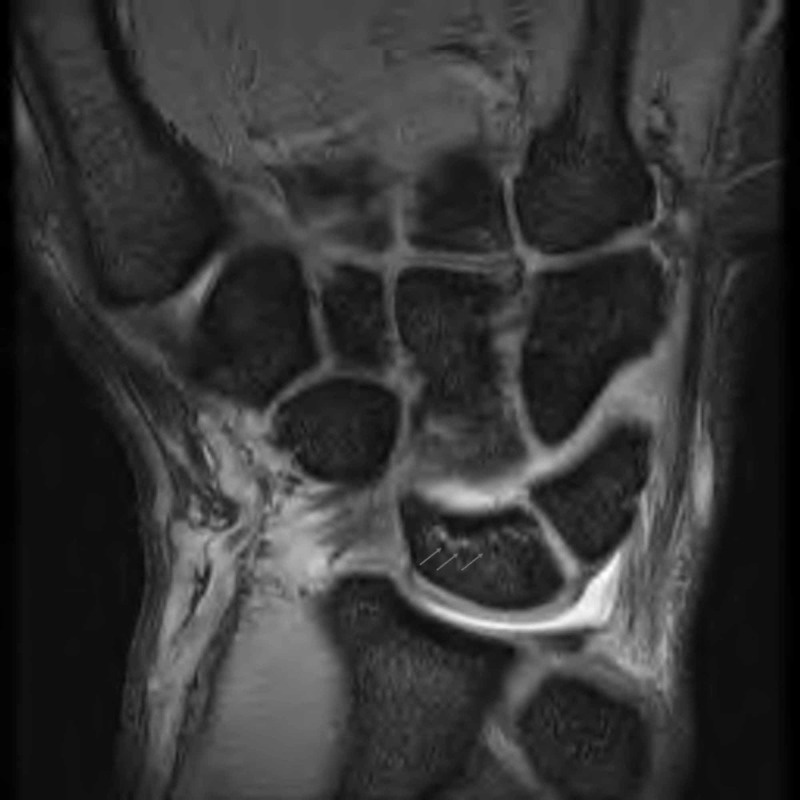
Coronal T2-weighted MR image showed mild increase in the signal intensity of the lunate bone.

**Figure 4:**
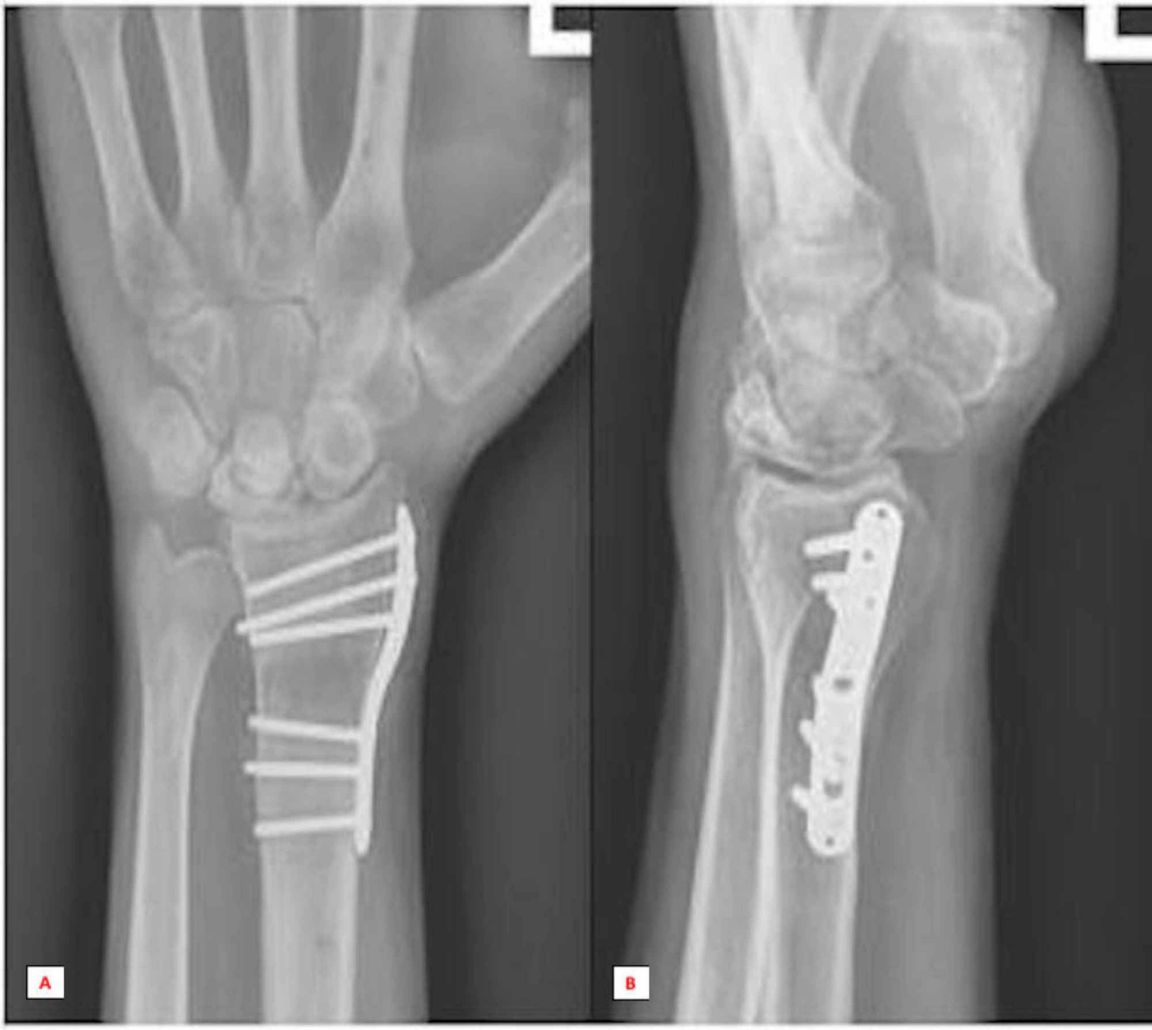
Anterior-posterior (A) and lateral (B) radiographic series at six months post-surgical follow-up revealed the complete collapse of the lunate.

A 28-year-old male presents with a two-month history of progressive left dorsal wrist pain at an outpatient clinic. There was no history of trauma, although the patient recalls the onset of his initial symptoms shortly after playing tennis. Physical examination showed tenderness over the left dorsal lunate, decreased wrist flexion-extension, radial deviation, and grip strength. PA radiographs of the left wrist in a neutral position revealed a negative UV with no other pathological findings (Figure 1). Two weeks later, subsequent MRI studies revealed a mild flattening of the lunate, a linear fissure fracture superiorly, decreased signal intensity of the lunate bone on a T1-weighted sequence (Figure 2) and an increased signal intensity on a T2-weighted sequence (Figure 3) suggestive of Kienböck’s disease, stage IIIA. The patient was initially treated with cast immobilization for four months for revascularization purposes. At follow-up, the patient remained symptomatic with no improvements in pain or function. Radiographic findings were indicative of the further collapse of the lunate which put into question the efficacy of future surgical operations in preventing end-stage disease. To continue with the care of the patient, he was presented with two options. One, allow the lunate bone to completely collapse and proceed with possible wrist arthrodesis to reduce wrist pain. Two, undergo surgical operation with the hopes of preventing the further collapse of the lunate. Given the presence of negative UV, the patient elected to undergo left radial shortening osteotomy with vascularized pedicle bone graft from the distal radius. Unfortunately, at the six-month post-surgical follow-up, radiographic series revealed the complete collapse of the lunate (Figure 4). However, at the 18-month post-surgical follow-up, the patient remains completely pain-free with the preserved grip strength of 60 pounds, and wrist flexion and extension of 65 and 50 degrees, respectively. Overall, the patient was satisfied with the outcome of the operation. Had he not elected to participate in the surgical operation, he would have remained symptomatic. More importantly, in the future, he would have most likely participated in wrist arthrodesis in order to reduce his pain at the cost of complete loss of range of motion. As a result, despite failure of surgical operation to prevent end-stage disease, the patient remains completely asymptomatic with no limitations to his daily living activities.

## Discussion

Kienböck’s disease is a rare condition characterized by avascular necrosis of the lunate, which results in the death of the bone. This condition commonly presents among individuals between the ages of 20 and 40 years [1]. This case presentation strengthens the idea that a complex interplay of repetitive microtrauma and anatomical variances may contribute to the pathogenesis of Kienböck’s disease [2]. Specifically, a history of repetitive microtrauma is emphasized after playing tennis resulted in the onset of the patient’s initial symptoms. Anatomically, the presence of negative UV and its contribution to the development of Kienböck’s disease is noted. The association between the presence of negative UV and subsequent development of Kienböck’s disease has been extensively analyzed, but its significance remains controversial given that the subset of patients present with neutral or positive UV [3,4]. Furthermore, negative UV is a common finding in a general population which is reflected in the research studies failing to confirm this relationship [13-17]. Nonetheless, this case presentation further adds to the positive association described in the previous studies [3,4]. Possible explanations for the differences seen in these results can be attributed to the inconsistent methods used to measure the UV, lack of investigator blindness and small sample sizes.

Furthermore, this report highlights the challenges of early diagnosis as disease presentation can be non-specific and mimic other common wrist pathologies such as sprained or fractured wrists. In addition, radiographic imaging can be inconclusive even during later stages of the disease, and their interpretations should be exercised with caution as false-negative findings can result in a high rate of misdiagnosis [6]. As a result, subtle diagnostic clues are essential for early intervention. In our case specifically, the presence of negative UV on the radiograph, and physical examination findings of persistent dorsal wrist pain, reduced grip strength, and range of motion warranted further investigation with an MRI. Given that treatment options are largely based on slowing the progression of the disease, a high index of suspicion and a subsequent MRI analysis are imperative for early diagnosis and treatment of patients with Kienböck’s disease.

Treatment of the condition is commonly directed by Lichtman’s classification system, which takes into account radiographic appearances to guide treatment (Table 1) [7].

**Table 1:** Lichtman’s classification system to guide treatment options for Kienböck’s disease.

Stages of the disease	Recommended treatment options
Stage I	Normal Radiographs. +/- linear fracture lines. MRI shows uniform signal decrease on T1-weighted images. Bone scan positive but nonspecific.
Stage II	Plain radiographs show lunate sclerosis, +/- fracture lines. No collapse of lunate
Stage IIIA	Lunate collapse with maintenance of carpal height and alignment
Stage IIIB	Lunate collapse plus any of the following: loss of carpal height, proximal capitate migration, flexed and rotated scaphoid
Stage IV	Stage IIIB + radiocarpal or midcarpal degenerative changes.

Current literature suggests that surgical interventions are required for stage IIIA [2]. It is important to note that both conservative and invasive procedures only slow the progression of the disease [8,17-19]. Therefore, it is a common practice to begin with cast immobilization prior to proceeding with any surgical operations. However, when comparing the efficacy of immobilization to surgery, specifically radial shortening osteotomy, the latter results in a better functional outcome. This is characterized by an improvement in pain, the range of motion and grip strength all of which were highlighted in this case presentation [17,18]. The advantage of radial shortening osteotomy can partly be explained by decreasing the mechanical stress on the lunate exerted by a relatively long radius [3,4]. Despite many treatment options available, current interventions have had limited success in reversing the progression of Kienböck’s disease until recently, where a case report demonstrated that hyperbaric oxygen therapy (HBOT), an off-label approach reversed stage IIIA in a 17-year-old male patient [1]. Originally, this conservative therapy is recommended for osteonecrosis of the femoral head with a type II indication defined by the 10th European Consensus Conference on Hyperbaric Medicine as the presence of sufficient evidence supporting its therapeutic action [20]. Therefore, for the initial treatment plan, HBOT as an adjunct therapy to cast immobilization may show promising results. In the future, additional research can evaluate the efficacy of HBOT in the treatment of Kienböck’s disease as this is the only conservative treatment to our knowledge that can drastically alter the clinical outcome of this entity.

## Conclusions

Kienböck’s disease is a rare condition that is characterized by avascular necrosis of the lunate bone. While limited knowledge into the pathogenesis of Kienböck’s disease exists, this report highlights the presence of repetitive microtrauma and negative ulnar variance as potential factors contributing to the development of this entity. Radiography is the initial imaging modality, but it is highly insensitive for early stages. Therefore, MRI should be considered in the care of patients after routine radiograph in those suspected of Kienböck disease is inconclusive. In our patient, the only diagnostic clues were the presence of negative UV on radiograph and persistent dorsal wrist pain with reduced range of motion that warranted further investigation with an MRI. Given that treatment options are largely based on slowing the progression of the disease, a high index of suspicion and subsequent MRI analysis are imperative for early diagnosis and treatment of Kienböck’s disease. Lastly, comparing the efficacy of conservative versus invasive treatment options, radial shortening osteotomy appeared to be superior which was highlighted by the patient’s complete lack of pain, some preserved range of motion and no limitations to his daily activities.
